# A2 Milk and BCM-7 Peptide as Emerging Parameters of Milk Quality

**DOI:** 10.3389/fnut.2022.842375

**Published:** 2022-04-27

**Authors:** Marzia Giribaldi, Cristina Lamberti, Simona Cirrincione, Maria Gabriella Giuffrida, Laura Cavallarin

**Affiliations:** Institute of Sciences of Food Production, National Research Council (CNR), Grugliasco, Italy

**Keywords:** beta-caseins, health effect, opioid peptides, technological traits, cows' milk

## Abstract

Beta-casein makes up about 30% of the total protein contained in milk and can be present in cows' milk in two distinct forms (A1 or A2) or as a combination of the two. The only difference between these two variants of β-casein (β-CN) is a single amino acid substitution. This results in a different behavior of the protein upon enzymatic cleavage, following human consumption or due to microbial action. In most of the commercially available milk containing A1 or A1/A2 β-CN variants, the β-casomorphin-7 peptide (BCM-7) is released upon digestion and during cheese manufacturing/ripening, while this does not happen with A2 milk. BCM-7 is a known μ-opioid receptor agonist that may influence the gastro-intestinal physiology directly and may also exert effects elsewhere in the body, such as on the cardiovascular, neurological and endocrine systems. The present article is aimed at a revision of prior review papers on the topic, with a focus on the impact of ingestion of A1 β-CN milk and A2 β-CN milk on any health-related outcomes and on the impact of A1 or A2 β-CN variant on technological properties of cows' milk. When systematic reviews were considered, it was possible to conclude that A2 β-CN exerts beneficial effects at the gastrointestinal level compared with A1 β-CN, but that there is no evidence of A1 β-CN having negative effects on human health. Physicochemical differences among cows' milk containing either β-CN A2 or β-CN A1 and their effects on technological properties are discussed.

## Introduction

Caseins constitute almost 80% of the proteins present in cows' milk. The casein class includes four different subtypes, α-s1-casein, α-s2-casein, κ-casein and β-casein (β-CN), which is one of the most abundant subtypes, accounting for about the 30% of the total caseins in cows' milk (1). The genetic characterization of dairy cows has highlighted that 13 genetic variants of β-CN exist: A1, A2, A3, B, C, D, E, F, G, H1, H2, I and J, among which the variants A1 and A2 are the most common in dairy cattle worldwide (2).

The difference in the two genetic forms concerns a single amino acid mutation, at position 67 of the polypeptide chain, which results in a histidine (His67) in A1 β-CN milk and a proline (Pro67) in A2 β-CN milk. The presence of His67 in A1 β-CN milk allows the human gastrointestinal enzymes, or the microbial proteolytic system, to perform a proteolytic cleavage of β-CN, which in turn causes the release of β-casomorphin-7 (BCM-7) during digestion and cheese ripening. On the other hand, proteolytic cleavage appears to be hampered in A2 β-CN, where Pro67 is present (2). β-casomorphins (BCMs) have been demonstrated to be able to bind the μ-opioid receptors that are located in the central nervous system and in the gastrointestinal tracts of humans (3). In the last few decades, the scientific community has shown increasing interest in the potential impact of BCM-7 and the related peptides on human health. In 2009, an EFSA scientific report concluded that the available data were insufficient to suggest a cause-effect relationship between BCMs and non-communicable disorders, such as cardiovascular disease, autism and insulin dependent diabetes mellitus (4). Four systematic reviews on A1 and A2 β-CN consumption and health-related outcomes have been conducted so far (1, 5–7). All of them concluded that the consumption of A2 β-CN milk may benefit the gastrointestinal status when compared to A1 β-CN milk, however the evidence about other health benefits was inconclusive (1, 5–7).

The possible effect of the differences in the structural and physicochemical properties of the main β-CN milk variants on specific technological aspects, and on cheese-making in particular, is a much less explored topic. The substitution of Pro/His at position 67 seems to affect the structure and conformation of the hydrophobic part of the β-CN sequence, and seems to ultimately affect the emulsifying and stabilizing properties of the two isoforms, as well as its coagulating capacity (8, 9).

The aim of the present review is to provide a critical concise overview of the available evidence: (i) on the health effects of consuming milk containing either A1 or A2 β-CN, and (ii) on the differences in the technological traits of milk containing A1 or A2 β-CN.

## Methodology

The present mini-review was conducted by resorting on different electronic databases (Scopus, Medline, WoS, PubMed and PMC), which were systematically searched on January 14th 2022 (Supplementary Figure 1). The search terms used in the Title/Abstract/Keywords (Scopus), or in the MeSH and Title/Abstract, or in the Topic (Medline and WoS), or in “All Fields” (PubMed and PMC), were: (casein AND “A2 milk” OR “A2A2 milk” OR “A2/A2 milk” OR “A2 beta casein” OR “A2 BCN” OR “A2 CASB” OR “A2 variant” OR “BCM7” OR “BCM-7”). The Document type was set as “Review”. The search results were first screened for relevance by considering the title and abstract by one of the authors (MG). In the case of ambiguity, the paper underwent a full-text analysis and was checked for final inclusion. The obtained results were then screened by another author (CL) to recognize studies included in the meta-review analysis of health-related outcomes associated with A2 milk and/or BCM-7 avoidance from the diet. The bibliographies of the studies included in the systematic review were also examined to find any additional pertinent studies. The adopted exclusion criteria were: the study design was a letter to the editor or a conference paper, or a study that had not been peer-reviewed; the review investigated non-bovine milk; the article merely described methods used for the detection of A2 β-CN or BCM-7; the review focused only on the prevalence of specific β-CN genotypes in different breeds.

The data extracted from each relevant review on clinical studies/ health-related aspects, reported in Table 1, included: the type of review considered (narrative or systematic), the type of study considered (human clinical or epidemiological studies, animal-based studies and *in vitro* studies), a summary of the main finding and the outcomes with a qualitative evaluation.

**Table 1:** List of the selected review papers reporting the association between of A1 or A2 β-casein consumption and health status.

	**Type of review**	**Type of studies considered**	**Main conclusion**	**Outcome qualitative evaluation**	**Outcomes**
Truswell (10)	Narrative	H, A	No association between A1 β-CN consumption and T1DM or CHD		
Bell et al. (11)	Narrative	H, A	A relationship between A1 milk and the risk of chronic condition such as T1DM and CVD has been shown. Moreover, A2 milk may be associated with less severe symptoms of autism and schizophrenia	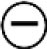	CVD + T1DM + ND
Kamiński et al. (2)	Narrative	H, A	Consumption of milk containing A2 β-CN associated with lower incidence of CVD and T1DM. SID, autism and schizophrenia possibly associated with high urine level of BCM-7	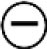	CVD + T1DM + SID+ ND
Raikos and Dassios (12)	Narrative	H, A, V	The formation of BCMs in infant formulas after simulated gastrointestinal digestion has been demonstrated. The *in vivo* effects of these peptides on the human physiology have not yet been fully described		
Pal et al. (13)	Narrative	H, A	In rodents, A1 milk increases GI transit time, inflammatory myeloperoxidase and dipeptidyl peptidase. In double-blind, randomized cross-over study, participants consuming A1 milk showed higher Bristol stool values compared with those receiving A2 milk	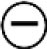	GI and T1DM
Chia et al. (14)	Narrative	H, A	In subjects with genetic risk factors, A1 β-CN and BCM-7 are causal triggers of T1DM.	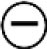	T1DM
Kalra and Dhingra (15)	Narrative	H	Exposure to A1/A1 milk may be linked with the rising incidence of T1DM in India.	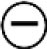	T1DM
Ledesma-Martínez et al. (16)	Narrative	A, V	Caseins and their fragments: (i) enhance lymphocytes proliferation and generation of antibodies; (ii) regulate normal hematopoiesis *in vitro* and *in vivo via* cytokine secretion, (iii) may induce apoptosis	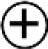	P on lymphocyte+ AP on neoplastic cells
Aslam et al. (17)	Narrative	H, A	BCM-7 might have multiple functions correlated to human health, but, the evidence is limited and mainly derived from either epidemiology, which cannot establish causality, or animal experiments, which may not be generalizable to humans	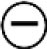	GI and obesity
Hegde (18)	Narrative	H, A	There was no clear evidence to link BCM-7 intake with T1DM, CVD or SID. Further clinical studies are needed to evaluate A1 milk effects in a broad range of population groups and dietary conditions		
Wong et al. (19)	Narrative	H, A, V	BCM-7: (i) may affect nervous, digestive and immune functions by altering the gene expression of μ-opioid receptors; (ii) seems to be associated with childhood mental disorders, T1DM, SID, AD.	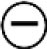	GI + ND + T1DM + SID +AD
Tulipano (20)	Narrative	H, A	BCM-7: (i) slows down GI transit time, (ii) induces a pro-inflammatory effect in the colon by opioid-receptor mediated mechanism	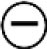	GI
Thiruvengadam et al. (21)	Narrative	H, A, V	A1 milk (i) consumption is a risk factor CVD, (ii) play a role in the onset of T1DM, iii) BCM-7 is present at high levels in blood, urine and CSF of children affected by autism, iv) depending on BCM-7 concentration, either suppression or stimulation of lymphocyte proliferation may occur, v) BCM-7 interaction with cancer cells seems to exhibit anti-cancerous activity *in vitro*	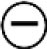 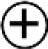	All
Kay et al. (22)	Narrative	H, A, V	Pro-inflammatory role of A1 β-CN on GI, endocrinological, neurological, and CV systems	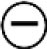	GI+ND+EC+CVD
Leischner et al. (23)	Narrative	H, A, V	BCM-7 seems to be able to induce apoptosis of pro-myeloic leukemia cells	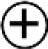	AP on neoplastic cells
Woodford (24)	Narrative	H, A, V	Food-derived opioids induce delayed GI transit, intestinal inflammation and permeability, altered microbiome	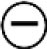	GI
Kohil et al. (25)	Narrative	H, A, V	Specific dietary patterns can exert a direct impact on pathogenesis of T1DM through epigenetic modifications. Among these, BCM-7 could act as an epigenetic modulator differentially methylate genes involved in T1DM development	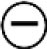	T1DM
Brooke-Taylor et al. (5)	Systematic	H, A, V	In rodents, A1 milk consumption causes delayed GI transit and increased inflammatory response. In humans, A1 milk consumption is associated with delayed GI transit, looser stool consistency and digestive discomfort (paper searching updated through April 2017)	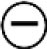	GI
Kuellenberg de Gaudry et al. (6)	Systematic	H	In humans, moderate certainty for adverse digestive health effects of A1 β-CN compared with A2 β-CN. Very low certainty for other health benefits (paper searching updated through October 2017)	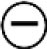	GI
Kuellenberg de Gaudry et al. (7)	Systematic	A	Considering animal studies, A2 milk might have beneficial effect on GI tract, whereas outcomes related to CVD and T1DM seem inconclusive (paper searching updated through March 2020)	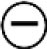	GI
Daniloski et al. (1)	Systematic	H, A	A2 β-CN can have some beneficial effects on the gastrointestinal system (paper searching updated through July 2020)	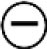	GI

*β-CN, β-casein; H, human clinical or epidemiological studies; A, animal-based studies; V, in vitro studies; T1DM, type 1 diabetes mellitus; CHD, coronary heart diseases; CV, cardiovascular; CVD, cardiovascular disorder; SID, sudden infant death syndrome; ND, neurological disorders; GI, gastrointestinal; BCM, β-casomorphin; AP/P, anti-proliferative/proliferative effect; EC, endocrinological effect; AD, atopic dermatitis. 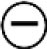 Negative health effect of A1 β-casein/ BCM-7 containing milk; 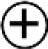 Positive health effect of A1 β-casein/ BCM-7 containing milk*.

The data extracted from each relevant review on qualitative/technological aspect, reported in Table 2, included: observed food product(s), investigated quality/ technological characteristic(s), and a summary of the main findings/evidences.

**Table 2:** List of the retrieved papers concerning physicochemical properties and protein functionality of A1 and A2 β-CN containing milk.

**Review**	**Matrix**	**Investigated quality/ technological characteristic(s)**	**Main findings**
Kamiński et al. (2)	Milk and dairy	Release BCM-7 during digestion	Release of BCM-7 following β-CN digestion requires a multi-enzyme system BCM precursors are detected in cheese; BCM-7 is largely detected in infant formulas A1 and A2 variants may present different physical properties in micelles
Raikos and Dassios (12)	Infant foods	Presence of BCM-7 in IF	BCM-7 and derivatives are released from commercial milk-based infant formulas following SGID
Nguyen et al. (9)	Milk and dairy	Presence of BCM-7 in dairy	BCM-7 is detected in raw milk and cheeses, but not in commercial yogurt, nor in pasteurized milk BCM precursors are detected in several cheeses BCM-7 in cheese does not originate from that originally present in milk, because peptides would be removed from the curd during the drainage of whey BCM-7 is higher in mold cheeses (Brie and Rokpol) than in semihard cheeses (Edamski, Gouda and Kasztelan) Proteolysis by enzymes from cheese starter cultures may reduce the amount of BCM-7
Pal et al. (13)	Milk and dairy	Presence of BCM-7 in dairy	BCM-7 is released from milk, yogurt and cheese following SGID Modest release of BCM-7 in cheese- and yogurt-making processes Certain yogurt bacteria may hydrolyze BCM-7
Brooke – Taylor et al. (5)	Milk	Release of BCM-7 during digestion	Release of BCM-7 following β-CN digestion requires a multi-enzyme system Presence of BCM-7 in fresh milk could be due to extended acidic hydrolysis *In vivo* studies indicate the release of BCM other than BCM-7
Gai et al. (8)	Milk and dairy	Effect of β-CN variants on milk yield, composition, protein structure, coagulation, foaming, emulsifying capacity	A2 variant has better chaperone capacity than A1, due to more proline helix formation Milk yield, protein yield, fat percentage and fat yield are significantly affected by β-CN variant, and the effect is enhanced by association with specific κ-CN variants A2 variant shows poorer coagulation properties than A1 (longer rennet coagulation time and looser curd), but may result in more easily digestible yogurt A2 variant shows a more acidic pI than A1, is more soluble, reaches the oil droplet surface more rapidly, but does not result in better emulsion stability. Results of the foaming properties of different β-CN variants are controversial
Summer et al. (26)	Milk and dairy	Presence of BCM-7 in dairy and effect on its release during digestion	Small amounts of BCM-7 could be released from A2 β-CN due to acidic hydrolysis in gastric environment BCM-7 is detected in a number of dairy products before and after SGID (pasteurized milk, cheese, laboratory-made yogurt, and other dairy products) Manufacturing and ripening in matured cheeses, and proteolysis by lactic acid bacteria and probiotics, exert a pivotal role in modulating the release BCM-7 before and after SGID The release of BCM-7 during SGID of sterilized infant formulas was hindered by protein glycation
Thiruvengadam et al. (21)	Milk and dairy	BCM-7 structure and metabolism	BCM-7 sequence is more hydrophobic, bitter to taste and its further digestion of is difficult because of the large percentage of proline BCM-7 and derivatives are released during the food processing (pasteurization, cheese- and yogurt-making, and other dairy products)

*IF, Infant Formula; BCM, β-casomorphin; β/κ-CN, Casein β/κ; SGID, Simulated GastroIntestinal Digestion*.

### A1 vs. A2 Cows' Milk β-Casein and Health

The review papers that reported evidence on the health effects of milk containing A1 and A2 β-CN, and which were selected using the meta review approach described above, are listed in Table 1.

The majority of the papers are reviews conducted without using a systematic approach, the so-called narrative reviews. They report several possible health effects linked to the consumption of milk containing A1 β-CN, ranging from non-communicable diseases to neurological and gastrointestinal disorders. The earlier revisions in the literature, which focused on the possible correlation between the cows' milk β-CN variant and an increased risk of developing specific diseases in humans, showed contradictory results. In 2005, Truswell (10) claimed that no association between A1 β-CN consumption and type I diabetes or coronary heart diseases could be demonstrated. On the other hand, in the same period, the reviews by Bell et al. (11) and Kamiński et al. (2), reported that the consumption of milk containing A1 β-CN seemed to be correlated with a higher incidence of cardiovascular disease and type 1 diabetes and high level of BCM-7 in urine might be associated with sudden infant death syndrome and neurological disorders, such as autism and schizophrenia. On the basis of the available evidence at that time, in 2009 EFSA concluded that “cause-effect relationship between the oral intake of BCM7 or related peptides and etiology or course of any suggested non-communicable diseases cannot be established” (4). Later on, in 2014, the review by Raikos and Dassios (12) concluded that BCM7 is released from infant formulas after simulated gastrointestinal digestion (SGID), but that the *in vivo* effects of these peptides on the human physiology cannot be fully described. In 2015, Pal et al. (13), reported that, in rodents, milk containing A1 β-CN significantly increases gastrointestinal transit time and the inflammatory marker myeloperoxidase. They also concluded that, despite the fact that clinical trials in humans were limited at that time, preliminary evidence from two human studies suggested proinflammatory factors alongside effects on gastrointestinal transit time. In 2017, Chia et al. (14) reviewed animal-based and *in vitro* evidence, concluding that A1 β-CN and BCM-7 were the dominant triggers of type 1 diabetes in individuals with genetic risk factors (14). This was in accordance to the observation of Kalra and Dhingra (15), who reported the hypothesis that exposure to A1/A1 β-CN milk of exotic breeds, such as those from India, might be linked with the rising incidence of type 1 diabetes. On the same topic, Kohil et al. (25) concluded that specific dietary patterns can exert a direct impact on pathogenesis of type 1 diabetes through epigenetic modifications and that BCM-7 could act as an epigenetic modulator, differentially methylating genes involved in type 1 diabetes development. However, more recently, both Aslam et al. (17) and Hegde (18) concluded that there was no clear evidence linking BCM-7 intake with type 1 diabetes, cardiovascular disease and neurological disorders. Aslam et al. (17), even considering the implications on obesity and gastrointestinal discomfort, stated that evidence was limited, and mainly derived from either epidemiology, which cannot establish causality, or animal experiments, which may not be generalizable to humans. When studies regarding the effect of purified/synthetized BCM peptides were reviewed, it was reported that they may improve the proliferation of lymphocytes and the generation of antibodies, while negatively regulate the proliferation of leukemia cells (16, 21). The anti-proliferative effect of BCM-7 has also been reported in a series of studies on prostatic cancer cells, breast cancer T47D cells and HL-60 promyeloic leukemia cells (23).

The ability of BCM-7 to pass through human intestinal barriers, and the consequent effects on immune functionalities have also been assessed (19). This is the mechanism potentially underlying the association of BCM-7 with several negative health outcomes, including type I diabetes, childhood mental disorders such as autism, the sudden infant death syndrome, and atopic dermatitis. One revision (20) has recently proposed a correlation between BCM-7 and digestive discomfort induced by a pro-inflammatory effect due to the same molecular mechanism in rodents. The suggested path is that of the activation of the opioid receptors in the gut, which could alter its microbial composition, with a subsequent impairment of the gut barrier integrity and bile acid metabolism. Woodford (24) suggested that the diverse presence of opioid receptors across all major human organs provides insights as to why the effects of A1 β-CN and BCM7 have also been identified in relation to such diversity of organs. This hypothesis has also been supported by a different series of studies (22, 26), in which the pro-inflammatory role of the A1 β-CN and its effect have been confirmed, not only at the gastrointestinal level, but also on the endocrinological, neurological and cardiovascular systems. Despite the large range of evidence on the effects of food- derived opioids on delayed intestinal transit, intestinal inflammation, intestinal permeability and an altered microbiome, it is clear that, when analyzing the conclusions of the narrative reviews, considerable variability in evidence exists regarding the effect on non-communicable diseases. The four systematic reviews published between 2017 and 2021 (1, 5–7) constitute a tool for interpreting such variability. These reviews were all conducted following the Preferred Reporting Items for Systematic Reviews and Meta-Analyses (PRISMA) checklist, with the aim of critically analyzing the existing data from well-controlled human clinical trials and animal studies on the impact of ingestion of A1 β-CN milk and A2 β-CN milk on health-related outcomes. The four systematic reviews concluded that consumption of A1 β-CN milk is associated with increased gastrointestinal transit times. As far as other health outcomes (CVD, diabetes, neurological diseases) are concerned, according to Kuellenberg de Gaudry and colleagues (6), the certainty of the evidence, according to the GRADE assessment, was judged as very low for most health outcomes considered in clinical trials and epidemiological studies published prior to October 2017. Using the same methodological approach, this was confirmed by Danilowsky and colleagues (1), for clinical trials and animal studies published prior to 2021 and for animal –based studies published prior to 2020 (7).

It can be, thus, concluded that, in order to unravel the possible role of particular β-CN variants and BCM forms in the development of non-communicable diseases, further longstanding clinical trials are needed with more participants from different geographical regions, gender, and age.

### The Physicochemical, Technological, and Functional Properties of A2 Variant of Cows' Milk β-CN

The bibliographic search highlighted that the majority of the published reviews scarcely considered the quality-related aspects associated with the A2/A2 genotype in milk and dairy products, or specific technological aspects, including cheese-making. When considered, the majority of the reviews (Table 2) reported the effect of processing/manufacturing techniques on the amount of BCM-7 released by food products containing either A1 or A2 β-CN. The only exception is represented by the very recent article by Gai et al. (8), that described in details most of the studies conducted until 2017 that were not addressing health-related aspects of A2 β-CN variant.

The amount and the generation of BCM-7 and related peptides during the manufacturing of different dairy products were revised multiple times in the past. BCM-7 was reported as detectable in a number of dairy products before and after SGID (milk, cheese, laboratory-made yogurt, and other dairy products) (9, 12, 13, 21, 26). BCM-7 in cheese was reported as not originating from that originally present in milk, since peptides would be removed from the curd during the drainage of whey (9). Also, manufacturing and ripening in matured cheeses, and proteolysis by lactic acid bacteria and probiotics, are thought to exert a pivotal role in modulating the release BCM-7 before and after SGID (26). For instance, BCM-7 was reported to be higher in mold cheeses (Brie and Rokpol) than in semi-hard cheeses, although proteolytic enzymes from cheese starter cultures were reported to reduce its amount during ripening (9). On the contrary, the occurrence of BCM-7 in yogurt has been rather controversial, and mainly reported for non-commercial, laboratory-scale yogurt manufacturing (9, 26), probably because of hydrolysis by specific yogurt bacteria (13). BCM-7 was largely detected in infant formulas (2), including some formulas produced by A2/A2 β-CN milk, thus indicating a possible problem of purity, in particular because of the presence of added proteins in the formulation (mainly whey protein concentrates and isolates), rather than being generated by processing itself. The release of BCM-7 during SGID of sterilized infant formulas was hindered by protein glycation (26).

The qualitative parameters revised by Gai et al. (8) included productive traits (milk yield and composition, coagulation attitude), as well as physical properties, of different variants of β-CN, including foaming, emulsifying and heat stability. The structural and conformational changes brought by the substitution of Pro/His in the β-CN sequence in A2 vs. A1 variant, indeed, occur in the hydrophobic part of the sequence, and may affect some technological aspects of the different isoforms, as emulsifying and stabilizing properties. For instance, the higher Pro content of A2 variant, increasing the formation of polyproline helices, seems to ultimately improve its chaperone ability.

Early reports on the milk quality traits in A2/A2 genotyped bovine breeds were mostly focused on productive traits, such as milk yield, protein and fat yields, and on the overall casein concentration. The β-CN A2 variant has been positively associated with higher milk yield and protein yield when compared to A1, while the contrary has been reported for fat percentage and yield (8). These characteristics may be enhanced by association of β-CN variants with specific κ-CN genotypes, such as for the composites A2A2-AB, A2A2-AA and A1A2-AE of β-κ-CN, positively correlated with milk and protein production, while variants A1A1-BB, A1A1-AB and A1A1-BE were associated with high fat percentage. More studies are required to better elucidate the effect of composite phenotypes or haplotypes on these aspects, and on the technological properties of milk, in order to possibly disclaim the overwhelming role of associated κ-CN variants.

One of the most frequently investigated aspect of A2/A2 milk was its poorer coagulating capacity than that of A1/A1 and A1/A2 β-CN milk (8). Several authors reported a higher occurrence of the A2 variant in non-coagulating milk, a longer coagulation time, looser curd formation, and a lower cheese yield. The reasons for this lower attitude toward rennet coagulation were mainly related to a lower exposed hydrophobicity, which results in more soluble isoforms, or in larger micelle size (2, 8). Indeed, the casein micelles in A2 milk were characterized by a larger particle size and lower negative ζ-potential, more random structures, and fewer α-helical structures than in A1 β-CN milk. The tendency to form looser curds may ultimately result in softer gels, characterized by larger pores, and a less dense protein network, also at the end of yogurt fermentation with A2/A2 β-CN milk. The resulting yogurt may therefore be more delicate and prone to deformation, but probably also more rapidly digested in the human stomach (8). For coagulation, the association of A2 β-CN variant in specific haplotypes with κ-CN also seems to result in higher occurrence of poorly or non-coagulating milk, as for A2A2-AA, A1A2-BE and A1A2-AE composite genotypes. Other technological aspects that were reported to be modified by specific β-CN variants were related to milk emulsifying and foaming properties. A2 variant has a more acidic pI than A1, and is therefore more soluble, reaching the oil droplet surface more rapidly. The presence of an additional proline in A2, as mentioned above, increases the content of polyproline-II helix, leading to a less ordered structure, that may influence the emulsifying properties and the foaming properties. Gai et al. (8) reported that the influence of β-CN A2 variant on foam formation and stability has been controversially reported in different studies, with poorer foaming capacity, or better foaming properties compared to A1, probably due to the different methods used in the formation of the foam.

## Conclusions

When analyzing the available evidence by means of a systematic approach, it is possible to conclude that consuming cows' milk containing A2 β-CN, instead of A1 β-CN, results in an overall improved gastrointestinal status and reduced milk related gut discomfort. As far as the technological traits are concerned, some differences have been observed among cows' milk containing either A2 β-CN or A1 β-CN, with A2 β-CN milk usually associated with poorer technological properties. The presence of an additional proline in A2 was reported to have a major impact on the hydrophobicity of the protein, thus leading to less ordered structures, that ultimately impact both casein micelle size, emulsifying and foaming properties, as well as the formation of rennet and acidic curd.

## Author Contributions

MGB: conceptualization, writing original draft, and formal analysis. CL: writing original draft and formal analysis. SC: writing original draft. MGF: writing review and editing. LC: conceptualization, writing review and editing, and supervision. All authors have contributed to the article and approved the submitted version.

## Conflict of Interest

MGB and LC declare financial interest, as previously funded by the Centrale del Latte di Italia S.p.A., within the frame of PROLAT project (funded by Regione Piemonte, Italy - POR FESR 2014-2020 - Bando Poli di Innovazione Asse I. Obiettivo specifico I.1b.1.-Azione I.1b.1.2, Call 2016). The remaining authors declare that the research was conducted in the absence of any commercial or financial relationships that could be construed as a potential conflict of interest.

## Publisher's Note

All claims expressed in this article are solely those of the authors and do not necessarily represent those of their affiliated organizations, or those of the publisher, the editors and the reviewers. Any product that may be evaluated in this article, or claim that may be made by its manufacturer, is not guaranteed or endorsed by the publisher.
