# Supplementary material for: A2 Milk and BCM-7 Peptide as Emerging Parameters of Milk Quality
Source: Front Nutr. 2022 Apr 27;9:842375. doi: 10.3389/fnut.2022.842375 (PMC9094626; doi:10.3389/fnut.2022.842375)
Supplement: Supplementary file 1 [file Image_1.pdf]

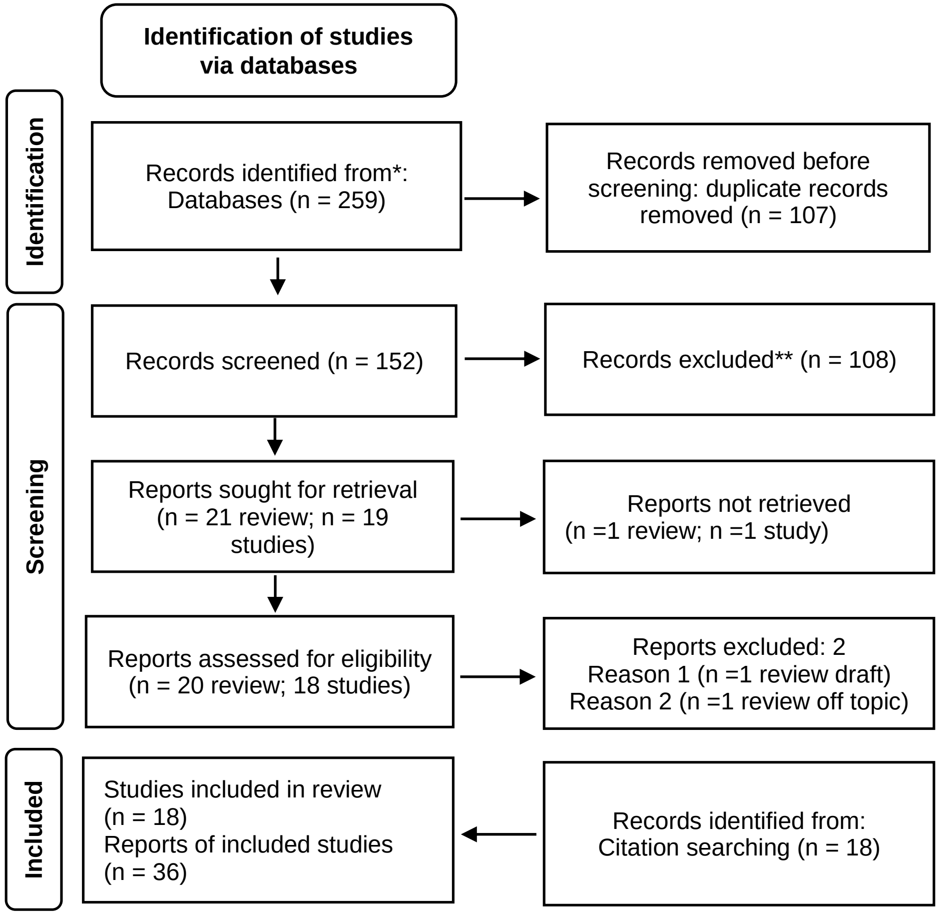

*Consider, if feasible to do so, reporting the number of records identified from each database or register searched (rather than the total number across all databases/registers).
**If automation tools were used, indicate how many records were excluded by a human and how many were excluded by automation tools.

From: Page MJ, McKenzie JE, Bossuyt PM, Boutron I, Hoffmann TC, Mulrow CD, et al. The PRISMA 2020 statement: an updated guideline for reporting systematic reviews. BMJ 2021;372:n71. doi: 10.1136/bmj.n71. For more information, visit: http://www.prisma-statement.org/
